# A general model for the motion of multivalent cargo interacting with substrates

**DOI:** 10.1098/rsif.2023.0510

**Published:** 2023-11-29

**Authors:** L. S. Mosby, A. Straube, M. Polin

**Affiliations:** ^1^ Centre for Mechanochemical Cell Biology & Division of Biomedical Sciences, Warwick Medical School, Coventry CV4 7AL, UK; ^2^ Physics Department, University of Warwick, Coventry CV4 7AL, UK; ^3^ Institute of Advanced Study, University of Warwick, Coventry CV4 7AL, UK; ^4^ Instituto Mediterráneo de Estudios Avanzados, IMEDEA, Esporles, Illes Balears 07190, Spain

**Keywords:** cargo transport, tip tracking, microtubules‌, multivalent cargo

## Abstract

Multivalent interactions are common in biology at many different length scales, and can result in the directional motion of multivalent cargo along substrates. Here, a general analytical model has been developed that can describe the directional motion of multivalent cargo as a response to position dependence in the binding and unbinding rates exhibited by their interaction sites. Cargo exhibit both an effective velocity, which acts in the direction of increasing cargo–substrate binding rate and decreasing cargo–substrate unbinding rate, and an effective diffusivity. This model can reproduce previously published experimental findings using only the binding and unbinding rate distributions of cargo interaction sites, and without any further parameter fitting. Extension of the cargo binding model to two dimensions reveals an effective velocity with the same properties as that derived for the one-dimensional case.

## 1. Introduction

Multivalent cargoes, defined by their simultaneous multi-site interactions with corresponding substrates, exist at a range of microscopic length scales and underpin a variety of interesting phenomena. Small multivalent ligand molecules, for example, exhibit diffusive motion on receptor-functionalized surfaces [1]; cytoskeletal polymers can interact and guide each other’s growth via multiple crosslinkers [2–5]; chromosomes are transported reliably towards cell poles during mitosis via multivalent interactions with microtubules [6,7]; and organelles like the endoplasmic reticulum can tubulate as a result of rapid interactions between their membrane-associated proteins and nearby polymerizing cytoskeletal filaments [8–10]. Most of these motility events involve microtubules acting as substrates, but they differ from the unidirectional stepping of molecular motors along microtubules since they are not coupled to ATP hydrolysis [11–13]. Instead, they are the result of the repeated binding and unbinding of individual interaction sites to a substrate, while they remain associated with the substrate due to their multivalency. These interactions can either be direct or mediated through additional factors. End-binding proteins (EBs), for example, bind transiently and preferentially to the unique tip structure at the growing ends of microtubules [14–21] and, although not individually transported by microtubules, their average population-level distribution follows polymerizing microtubule tips [2,10,20–26]. Multivalent cargo can then co-move with the growing microtubule end by interacting simultaneously with multiple EBs while maintaining at least one cargo–EB–microtubule linkage at all times [5,27].

Despite their differences, the examples above share several fundamental characteristics. Single-site cargo–substrate interactions are short-lived [1,5,27,28]; the average number of interaction sites will strongly influence the mean dwell times of cargo [7,28–30]; and relatively few interaction sites are required to generate cargo motion [1,28]. We propose that the underlying mechanism by which the valency of cargo is coupled to its resulting dynamics is fundamentally the same for all of these systems. Unfortunately, there is currently no general model of multivalent cargo transport that can describe the behaviour of all of these systems.

Traditionally, the dynamics of multivalent cargo are modelled by studying the discrete binding dynamics of their individual interaction sites [1,5,27–31], but combinatorial complexity often results in the need for numerical solvers [32] or direct simulations [1,4,27]. Some aspects of cargo motion can be derived analytically from either the forces associated with cargo–substrate linkages [30,31], or the effective free energies associated with the possible binding states of cargo [5]. The latter model has been used to begin investigating the transport of multivalent cargo resulting from position dependence in cargo–substrate interactions, although this is currently limited to step-like binding/unbinding rate distributions in simplified interaction networks [5]. Using these approaches, it is difficult to derive intuitive analytical formulae for the coarse-grained motion of cargo directly from their microscopic interactions. One alternative method is to assume the form of effective coarse-grained dynamical variables and to fit parameters using experimental results. This heuristic procedure is useful as a description, but misses the link to any actual microscopic mechanisms.

Here, we develop a general model for multivalent cargo motion that only requires the binding and unbinding rate distributions of individual interaction sites as input parameters. This model allows quantitative predictions of coarse-grained cargo dynamics to be made without parameter fitting. We explicitly derive the equations describing the motion of multivalent cargo, and show that their coarse-grained dynamics are diffusive if cargo–substrate interaction rates are homogeneous, while position-dependent binding/unbinding leads to net cargo transport. The analytical form of the coarse-grained velocity agrees well with the results of stochastic cargo binding simulations, and can be used to directly show that cargo accumulate at regions of increased cargo–substrate binding rate and decreased cargo–substrate unbinding rate.

Our results show that cargo dynamics are strongly dependent on the number of interaction sites on the cargo surface: cargoes with a small number of interaction sites exhibit dwell times too small to generate meaningful motion while bound. By increasing the number of interaction sites the motion becomes governed by both an effective velocity and diffusivity. A further increase results in approximately deterministic cargo motion due to the damping of stochastic effects. Analytical and numerical results also suggest that the spatial distribution of substrate-bound cargo interaction sites strongly influences the cargo’s ability to diffuse on a substrate. In order to test our results in biologically relevant conditions, we consider the EB-mediated processive transport of cargo and show that the model is able to reproduce physiological tip-tracking starting exclusively from EB–microtubule attachment–detachment rates estimated from published work. The model is then expanded to two dimensions with the aim of describing more complex biological systems. We conclude with a broadly accessible summary of the key points of the work. We believe that the general approach developed here will be valid for any binding/unbinding rate distribution profiles and for arbitrarily complex interaction networks.

## 2. Results

### 2.1. The cargo binding model

**Figure 1.**
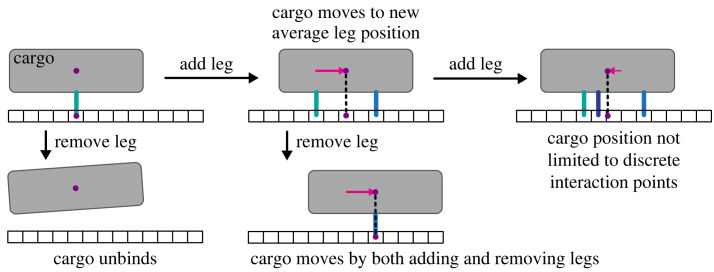
Cargo binding model. An individual cargo initially binds with a single leg (interaction point), and then either new legs bind or previously bound legs unbind according to the binding or unbinding rates. Following each binding/unbinding event, the cargo position is updated to be equal to the average bound leg position. Interaction with the substrate ceases upon unbinding of the last leg.

We posit that the motion of a substrate-bound multivalent cargo originates from the ability of its interaction sites (henceforth referred to as legs) to repeatedly bind/unbind at different positions on the substrate (figure 1). This implies that this process should be able to explain both the diffusive motion of multivalent ligand molecules interacting with receptor-functionalized surfaces [1] and the processive motion of multivalent cellular components interacting with cytoskeletal polymers [5,7,27,28,31]. Differences in cargo transport would then reflect differences in the spatial distribution of leg–substrate interaction rates.

In between binding/unbinding events, the instantaneous position of the cargo will be determined by a balance of forces. For a cargo of length *L* with *N* legs, of which *n* are bound at positions {*x*_*l*_(*t*)} at time *t*, we will define the centre position *x*_*a*_(*t*) as the average of the bound leg positions, such that $\sum_{l=1}^{n}(x_{l}(t)-x_{a}(t))=0$. Although this assumption can be generalized, it enforces a simple force equilibrium criterion on the cargo. Each cargo initially binds in the *n* = 1 state and then unbinds once *n* = 0, such that its average dwell time while bound to the substrate, *t*_dwell_(*x*), can be calculated using previously published results [29,30] (see electronic supplementary material, figure S1). Individual legs can bind to (*m* = 1) or unbind from (*m* = 2) the substrate with position-dependent rates *κ*_*m*_(*x*). However, these rates need to be modified to take into account the size and shape of the cargo. We do this by including two probability distributions *S*(*x*|*x*_*a*_(*t*), *n*) and *P*_*b*_(*x*|*x*_*a*_(*t*), *n*). The ‘shape factor’ *S*(*x*|*x*_*a*_(*t*), *n*) is the probability distribution function that describes the probability of one of the cargo’s *N* − *n* unbound legs binding at the position *x* when the cargo is at *x*_*a*_(*t*). This modifies the binding rate to *k*_1_(*x*|*x*_*a*_(*t*), *n*) = *κ*_1_(*x*)*S*(*x*|*x*_*a*_(*t*), *n*), thereby extending the concept of local concentration of substrate binding sites previously introduced in [33]. For example, a shape factor *S*_*H*_(*x*|*x*_*a*_(*t*)) = (*θ*(*x* − (*x*_*a*_(*t*) − *L*)) − *θ*(*x* − (*x*_*a*_(*t*) + *L*))/2*L* (where *θ*(*x*) is the Heaviside step function) states that unbound cargo legs can bind to the substrate uniformly within the range *x*_*a*_(*t*) − *L* ≤ *x* ≤ *x*_*a*_(*t*) + *L*, defining a putative cargo width of 2*L*. This approach assumes that legs cannot interact with each other, and that once unbound they can access and try to bind to any position on the substrate allowed by the shape factor, regardless of the time elapsed since their last unbinding. The latter condition requires the typical binding timescale ($t_{b}∼1/\max(\kappa_{1}(x))$) to be much larger than the timescale associated with the diffusion of unbound legs on the cargo surface (*t*_*d*_ ∼ (2*L*)^2^/*D*_*l*_; *D*_*l*_ is the effective diffusivity of the cargo leg). If needed, these simplifying assumptions can be relaxed and a more complex, possibly time-dependent, shape factor can be introduced to modulate the legs’ distribution around the centre of the cargo *x*_*a*_(*t*).

**Figure 2.**
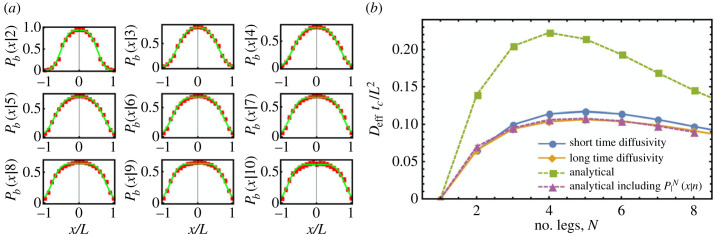
Dynamics of cargo with uniform binding and unbinding rates. (*a*) Example bound leg distributions (red) for simulated 10-legged cargo with position-independent binding/unbinding rates, averaged over cargo centre position (10 000 simulated). Fits (green) were calculated as described in electronic supplementary material, §6. (*b*) Cargo exhibit distinct short- and long-time diffusivities. Distributions from simulations were calculated from the gradients of mean-squared displacement (MSD) distributions, as shown in electronic supplementary material, figure S2 (100 000 simulated for 1 ≤ *N* ≤ 7; 50 000 simulated for *N* = 8; 25 000 simulated for *N* = 9; 10 000 simulated for *N* = 10), and analytical distributions were obtained using equations (2.1), (2.2) and (2.5). Errors cannot be calculated for the analytical distribution that does not require bound leg distributions, since the motion is purely deterministic. Error bars for other distributions are too small to see.

Since cargo legs are stationary once bound, unbinding events can only occur from positions within the set {*x*_*l*_(*t*)} (henceforth lengths will be understood to be in units of *L*). This requirement is simple to implement computationally, but complex to include analytically. Therefore, to make analytical progress, we tackle this problem at the mean-field level by introducing the coarse-grained bound leg distribution *P*_*b*_(*x*|*x*_*a*_(*t*), *n*). This probability distribution function describes the probability of one of the cargo’s *n* bound legs being at position *x* when the cargo is at *x*_*a*_(*t*), and it modifies the unbinding rate distribution such that on average *k*_2_(*x*|*x*_*a*_(*t*), *n*) = *κ*_2_(*x*)*P*_*b*_(*x*|*x*_*a*_(*t*), *n*). The bound leg distribution is clearly linked to the cargo’s shape factor. For example, if the shape factor only permits binding of cargo legs within a specified range of positions, then the bound leg distribution will decay quickly at the extremities of this range (as will be shown in figure 2*a*).

In the presence of position-dependent leg binding and unbinding rates *κ*_1,2_(*x*), a cargo will exhibit position-dependent average displacements and event rates. The *i*th moment of the cargo displacement distribution, $\lambda_{m}^{\left(i\right)}(x_{a}(t),n)$, and the average rate, ${\bar{k}}_{m}(x_{a}(t),n)$, corresponding to each type of event (*m* = 1, 2) are given by (see electronic supplementary material, §1)

2.1 $$\lambda_{m}^{\left(i\right)}(x_{a}(t),n)={\left(\frac{1}{n+\Delta_{m}}\right)}^{i}\left(\frac{\int {\left[\Delta_{m}(x-x_{a}(t))\right]}^{i}k_{m}(x|x_{a}(t),n)\;dx}{\int k_{m}(x|x_{a}(t),n)\;dx}\right)$$

and

2.2 $${\bar{k}}_{m}(x_{a}(t),n)=(N\delta_{m,1}-n\Delta_{m})\int k_{m}(x|x_{a}(t),n)\;dx,$$

where Δ_*m*_ = *δ*_*m*,1_ − *δ*_*m*,2_ ≡ 3 − 2*m* and *δ*_*i*,*j*_ is the Kronecker delta. The second term in equation (2.1) calculates the mean *i*th power of the difference between the positions of the binding or unbinding event and the cargo centre, with the factor ∝(1/(*n* + Δ_*m*_))^*i*^ accounting for the smaller displacements of the cargo centre position when more legs are bound. Instead, equation (2.2) is the simple multiplication of the average rate of binding/unbinding over the extent of the cargo by the number of legs available for each type of transition.

We then introduce a third type of transition (*m* = 3) to take into account any deterministic motion of the cargo independent of position and *n*. This can be used, for example, to model cargo that bind to substrates that themselves can move [7,28,31], or cargo motion in the co-moving frame of a growing substrate such as the polymerizing tip of a microtubule. This motion can be introduced analytically by assuming that all cargo legs are displaced by Δ*x* (resulting in $\lambda_{3}^{\left(i\right)}={\left(\Delta x\right)}^{i}$) at a rate ${\bar{k}}_{3}$, and can be generalized to accept different displacement and wait-time distributions.

Armed with these quantities, it is possible to derive the continuity equation satisfied at the mean-field level by *P*(*x*, *t*), the probability distribution function of finding cargo at the position *x* at time *t* [34]. A truncated Kramers–Moyal expansion leads to the Fokker–Planck equation (see electronic supplementary material, §2):

2.3 $$\begin{array}{rl} \frac{\partial P(x,t)}{\partial t} & =\frac{\partial }{\partial x}\left[D_{\mathrm{eff}}(x)\frac{\partial P(x,t)}{\partial x}\right]-\frac{\partial }{\partial x}\left[v_{\mathrm{eff}}(x)P(x,t)\right]\\ & \;+k_{\mathrm{on}}(x)-k_{\mathrm{off}}(x), \end{array}$$

where *k*_on_(*x*) and *k*_off_(*x*) are the position-dependent effective binding and unbinding rates (respectively) of cargo modelled as single, composite bodies. This effective unbinding rate is equivalent to the reciprocal of the average dwell time of the cargo (see electronic supplementary material, §3). The position-dependent effective velocity and diffusivity, *v*_eff_(*x*) and *D*_eff_(*x*), are defined as

2.4 $$\begin{array}{r} v_{\mathrm{eff}}(x)=\;\sum_{n=1}^{N}\left[P_{n}(x)\sum_{m=1}^{M}\left({\bar{k}}_{m}(x,n)\;\lambda_{m}^{\left(1\right)}(x,n)\right)\right]-\frac{\partial D_{\mathrm{eff}}(x)}{\partial x}\\ =\;\;S_{\lambda }^{\left(1\right)}(x)-\frac{\partial D_{\mathrm{eff}}(x)}{\partial x} \end{array}$$

and

2.5 $$\begin{array}{rl} D_{\mathrm{eff}}(x) & =\left(\frac{1}{2}\right)\sum_{n=1}^{N}\left[P_{n}(x)\sum_{m=1}^{M}({\bar{k}}_{m}(x,n)\;\lambda_{m}^{\left(2\right)}(x,n))\right]\\ & =\frac{S_{\lambda }^{\left(2\right)}(x)}{2}, \end{array}$$

where *P*_*n*_(*x*) is the probability of cargo having *n* legs bound at position *x* (see electronic supplementary material, §3; not to be confused with the bound leg distribution), and *M* = 3 for a system with binding/unbinding events and a net velocity. Together, equations (2.3)–(2.5) predict that cargo can exhibit both diffusive and processive motion depending on the forms of the cargo–substrate interaction rates *κ*_*m*_(*x*).

The terms $S_{\lambda }^{\left(1,2\right)}(x)$ defined in equations (2.4) and (2.5) both consist of sums over independent contributions from *M* different types of events, averaged over the expected number of bound legs at each position. They can be understood as the product of a position-dependent total event rate and a suitably weighted average of the displacement moments $\lambda_{m}^{\left(1,2\right)}$ (see electronic supplementary material, §2). Substituting equations (2.1) and (2.2) into equation (2.4), we see that the direction of $S_{\lambda }^{\left(1\right)}(x)$ depends on $\pm \left[\int_{x_{a}}|x-x_{a}|k_{m}(x|x_{a},n)\;dx-\int^{x_{a}}|x-x_{a}|k_{m}(x|x_{a},n)\;dx\right]$, which describes the difference in the binding/unbinding rate distributions (± respectively) averaged over the two halves of the cargo width before and after the midpoint *x*_*a*_. For slowly varying binding/unbinding rates, this has the appealing intuitive interpretation that $S_{\lambda }^{\left(1\right)}(x)$ acts in the direction of increasing binding rate (∝*k*_1_′(*x*|*x*_*a*_(*t*), *n*), where ′ indicates a derivative with respect to *x*) and decreasing unbinding rate (∝−*k*_2_′(*x*|*x*_*a*_(*t*), *n*); see §2.3).

This coarse-grained description of cargo motion at the population level can be accompanied by a Langevin equation for the stochastic trajectories *x*_*a*_(*t*) of individual cargo (see electronic supplementary material, §5):

2.6 $$dx_{a}(t)=S_{\lambda }^{\left(1\right)}(x_{a}(t))\;dt+\sqrt{S_{\lambda }^{\left(2\right)}(x_{a}(t))}\;dW(t).$$

Here, d*W*(*t*) is the standard Wiener process [35] and the multiplicative noise is intended in the Itô sense. The first term in equation (2.6) defines the effective deterministic motion of a bound cargo due to gradients in the binding and unbinding rates of its legs, while the second term describes the noise affecting its motion. This means that the fixed points of the dynamics will occur at positions x* where $S_{\lambda }^{\left(1\right)}(x^{*})=0$, meaning that

2.7 $$\begin{array}{rl} & {\left[\sum_{n=1}^{N}P_{n}(x)({\bar{k}}_{1}(x,n)\;\lambda_{1}^{\left(1\right)}(x,n)+{\bar{k}}_{2}(x,n)\;\lambda_{2}^{\left(1\right)}(x,n))\right]|}_{x=x^{*}}\\ & \;=-{\bar{k}}_{3}\;\Delta x. \end{array}$$

For example, for EB-mediated cargo transport in the rest frame of a microtubule growing with velocity *v*_MT_, $-{\bar{k}}_{3}\Delta x=v_{\mathrm{MT}}$, and a fixed point will then arise when the effective velocity generated by the binding/unbinding dynamics of cargo legs—the left-hand side of equation (2.7)—is equal to *v*_MT_. Fixed points will be stable when ${\left(\partial S_{\lambda }^{\left(1\right)}(x)/\partial x\right)|}_{x=x^{*}}<0$, and will then correspond to the positions where cargo accumulate. In the previous example, this would correspond to the cargo tracking the polymerizing microtubule tip.

### 2.2. Probing cargo dynamics

Having defined the model, we can start probing cargo dynamics by comparing the more intuitive analytical solution with a numerical one. For simplicity, in the following sections, we will assume the uniform shape factor *S*_*H*_(*x*|*x*_*a*_(*t*)). The bound leg distributions *P*_*b*_(*x*|*x*_*a*_(*t*), *n*) cannot be derived analytically in general, but they are straightforward to obtain from stochastic cargo binding simulations. Example bound leg distributions for use in equations (2.1) and (2.2) are shown in figure 2*a* and electronic supplementary material, figure S4*a*, together with a heuristic fit which provides a parametric family of analytical curves that can be used in the integrals (see electronic supplementary material, §6). For uniform binding/unbinding rates (*κ*_1_ = *κ*_2_) and ${\bar{k}}_{3}=0$, figure 2*a* shows that bound leg distributions are symmetric, and equations (2.1), (2.4) and (2.5) immediately imply that $\lambda_{m}^{\left(1\right)}(x_{a}(t),n)=v_{\mathrm{eff}}(x)=0$. The cargo should then exhibit diffusive motion with no net drift, in agreement with stochastic simulations (see electronic supplementary material, figure S2*a*).

Interestingly, for *N* > 2, simulated cargo exhibit distinct short- and long-time diffusivities as a result of their binding dynamics (see figure 2*b*; electronic supplementary material, figure S2; for 3 ≤ *N* ≤ 10 each pair of the short- and long-time diffusivities are separated by at least $10\max(\zeta_{\mathrm{short}},\zeta_{\mathrm{long}})$, where *ζ*_short,long_ are the errors associated with the short- and long-time diffusivities in figure 2*b* and electronic supplementary material, figure S2, respectively). Cargo always initially bind in the *n* = 1 state and, for those that survive long enough, the probability *P*_*n*_(*x*) of having *n* legs bound will take some time to relax to the steady-state form (see electronic supplementary material, figure S3). Given that the MSD due to binding/unbinding events depends on the average number of bound legs (for example equation (2.1) shows that $\lambda_{1}^{\left(2\right)}(x_{a}(t),n)\propto 1/{\left(n+1\right)}^{2}$), the diffusivity measured a short time after initial attachment is expected to be higher than that measured later for longer living cargo. Intuitively, the displacement caused by the binding or unbinding of individual legs will be larger when the number of bound legs is small. Figure 2*b* and electronic supplementary material, figure S2, show that this is indeed observed in simulations, and we propose that it could also explain previously published experimental MSD data showing multivalent cargo diffusivities that decrease as a function of time spent bound [5]. The definition of short and long time is relative to a ‘separation timescale’ $≲t_{c}=1/\kappa_{2}$ for *N* > 2 (see the crossover point in electronic supplementary material, figure S2*b*, and the plateau in electronic supplementary material, figure S3). This separation timescale diverges at *N* = 2, such that the short- and long-time diffusivities coincide in figure 2*b*. Figure 2*b* also shows that the analytical model defined in equations (2.1), (2.2) and (2.5) generates effective diffusivities in agreement with the long-time values obtained from simulations, but only after introducing the steady-state bound leg distributions shown in figure 2*a*. Excluding the bound leg distributions instead leads to a higher effective diffusivity due to the overestimation of the probability of finding bound legs at the extremities of the cargo. Finally, the diffusivities in figure 2*b* are non-monotonic functions of the number of cargo legs. This is the result of competition between a total rate of events which increases with increasing *N* (leading to a higher rate of change in cargo position), and an average displacement per event which scales as ∼1/(*n* ± 1)^2^ (see equations (2.1) and (2.2)).

In order to explore the effect of spatially varying cargo–substrate interactions, let us now consider a binding rate distribution of the form

**Figure 3.**
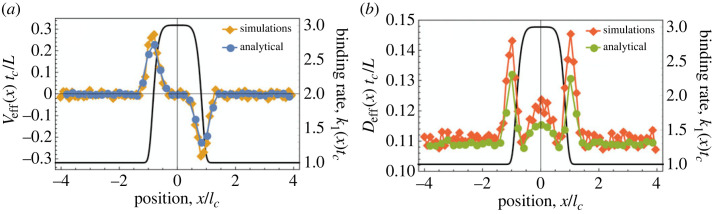
Dynamics of cargo with position-dependent binding and unbinding rates. (*a*) Effective velocities and (*b*) effective diffusivities, derived by substituting the results of simulations (orange, red; 250 000 simulated) or equations (2.1) and (2.2) (blue, green) into equation (2.4) or equation (2.5), respectively. The corresponding position-dependent binding rate distribution defined in equation (2.8) is also shown (black). Both effective velocity distributions show that cargo are ‘attracted’ towards the central region of increased cargo–substrate binding rate defined in equation (2.8). Distribution smoothing has been outlined in the electronic supplementary material, Methods, and only every fifth data point of each distribution is shown for clarity. Error bars are too small to see.

2.8 $$\kappa_{1}(x)t_{c}=\left(\frac{4}{1+\exp({\left(x/2\right)}^{6})}\right)+1,$$

for which we define the characteristic length scale *l*_*c*_ = 2(ln(39))^1/6^ ≃ 2.5 as the distance from the origin to the position where *κ*_1_(*x*) = 1.1*κ*_2_. This distribution has a central region with higher binding rate than its surroundings (figure 3). As we can see in figure 3*a*, the result of a binding rate distribution given by equation (2.8) and a position-independent unbinding rate is that cargo exhibit an effective velocity profile that acts to accumulate the cargo near the centre of the high-binding-rate region. This is also true when ${\bar{k}}_{3}\Delta x\neq 0$, in which case the stable fixed point for bound cargo motion is shifted in the direction of Δ*x*, as predicted by equation (2.7) (see electronic supplementary material, figure S6). Figure 3 shows that the effective velocity and diffusivity calculated from equations (2.1), (2.2), (2.4) and (2.5) compare very well with the results from full numerical simulations. The small discrepancies are the result of the mean-field analytical approach using the steady-state probability of cargo having *n* legs bound, *P*_*n*_(*x*), which is only correct for long-lived cargo. We find similarly good agreement between bound leg distributions from simulations and their corresponding heuristic fits (see electronic supplementary material, §6 and figure S4*a*).

**Figure 4.**
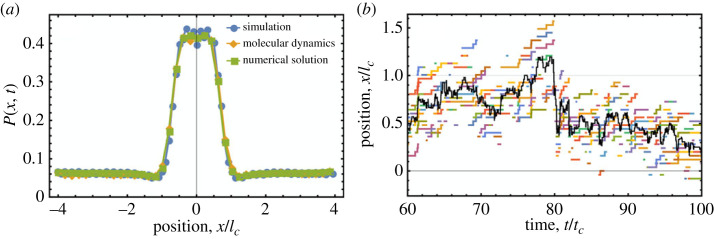
Average cargo behaviour. (*a*) The PDF *P*(*x*, *t*) describing the probability of finding 4-legged cargo at the position *x* averaged over all simulation time *t* exhibits a peak in the central region when cargo exhibit the binding rate defined in equation (2.8) and a uniform unbinding rate. Stochastic simulation results (250 000 simulated) agree with those obtained using molecular dynamics simulations (d*t* = 0.1, *t*_max_ = 1 000 000) and by numerically solving equation (2.3) using an analytically derived effective velocity distribution. Only every fifth data point of each distribution is shown for clarity, and error bars for stochastic simulation data are too small to see. (*b*) Section of an individual 10-legged cargo track (black) showing the positions of each leg while bound (coloured) when subject to ${\bar{k}}_{3}\Delta x=0.1L/t_{c}$. Cargo legs that unbind near *x* = *l*_*c*_ preferentially rebind within the central region of increased cargo–substrate binding rate defined by equation (2.8) (see displacements near *t*/*t*_*c*_ ≃ 80).

Direct numerical simulations of cargo that interact with a substrate via the binding rate profile in equation (2.8) and a uniform unbinding rate (see figure 3) result in a probability distribution function *P*(*x*, *t*) that exhibits a strong central peak at sufficiently long times (figure 4*a*). The tracks of individual cargo clearly show that this is the result of the preferential binding of cargo legs within the central high-binding-rate region (figure 4*b*). Figure 4*a* also shows the probability distributions obtained by feeding the functions *v*_eff_(*x*) and *D*_eff_(*x*) (equations (2.4) and (2.5) and figure 3) into either the continuum model in equation (2.3), or stochastic molecular dynamics simulations of the Langevin dynamics in equation (2.6). These effective descriptions of cargo dynamics lead to probability distributions that agree remarkably well with the full model, while providing a simpler and more intuitive understanding of the behaviour of cargo. This is also true for the case where ${\bar{k}}_{3}\Delta x\neq 0$, for which the distribution *P*(*x*, *t*) is skewed in the direction of Δ*x* (electronic supplementary material, figure S6).

### 2.3. Behaviour of the effective velocity

When the cargo is sufficiently small compared to the characteristic lengths of variation in *κ*_1,2_(*x*) (i.e. *L* ≪ |*κ*_1,2_′(*x*_*a*_(*t*))/*κ*_1,2_″(*x*_*a*_(*t*))|), the explicit dependence of $S_{\lambda }^{\left(1\right)}(x)$ on the underlying rate distributions can be simplified by using a leading order approximation to *κ*_1,2_(*x*). Assuming a shape factor *S*_*H*_( · ), this results in

2.9 $$S_{\lambda }^{\left(1\right)}(x)≃\sum_{n=1}^{N}\left[P_{n}(x)\left(\left(\frac{(N-n)L^{2}}{3(n+1)}\right)\kappa_{1}^{\prime }(x)-\left(\frac{n}{n-1}\right)\kappa_{2}(x)\;I(x,n)\right)\right]+{\bar{k}}_{3}\;\Delta x,$$

where $I(x,n)=\int (y-x)P_{b}(y|x,n)\;dy$ is the average difference between the unbinding position of a cargo leg and the cargo centre. We note that the distribution *P*_*b*_(*y*|*x*, *n*) is symmetric only for uniform binding/unbinding rates, in which case $S_{\lambda }^{\left(1\right)}={\bar{k}}_{3}\Delta x$. For non-uniform binding/unbinding rates, the first term in equation (2.9) predicts that the component of the effective velocity due to binding events is proportional to the local gradient in the binding rate distribution, whereas the second term states that the component due to unbinding events is dominated by variations in the bound leg distribution. For the cargo–substrate interaction rates defined in equation (2.8), it can be shown that the first term dominates over the second (see electronic supplementary material, figures S5 and S6*c*). In this case, equation (2.9) predicts, for ${\bar{k}}_{3}\Delta x=0$, that $S_{\lambda }^{\left(1\right)}(x)\propto L^{2}\kappa_{1}^{\prime }(x)$, as well as that $S_{\lambda }^{\left(1\right)}(x)$ increases monotonically as a function of *N* until it plateaus at the value $\lim_{N\to \infty }(S_{\lambda }^{\left(1\right)}(x))=L^{2}\;\kappa_{1}^{\prime }(x)/3$ (see electronic supplementary material, §4). Equation (2.9) also predicts that cargo will accumulate around the maximum in the binding rate distribution. However, when ${\bar{k}}_{3}\Delta x\neq 0$ the stable fixed point around which cargo accumulates shifts from the maximum of the binding rate towards one of the peaks in *κ*_1_′(*x*), depending on the sign of Δ*x*.

It is also possible for cargo to overhang the edges of a non-periodic substrate, in which case they will effectively experience step-like changes in their binding and unbinding rates (*κ*_1,2_(*x*) = 0 beyond the edge) and *L* will no longer be small compared to the characteristic lengths of variation in *κ*_1,2_(*x*) near the boundary. The dynamics of these cargo will still be described by equations (2.1), (2.2), (2.4) and (2.5), but no longer by equation (2.9). For binding/unbinding rates *κ*_1,2_ which are uniform within the bounds of the substrate, the formalism in equations (2.1), (2.2) and (2.4) can be used to show that there will be a contribution to the effective velocity of the cargo of the form

2.10 $$\begin{array}{rl} S_{\lambda }^{\left(1\right)}(x) & =\sum_{n=1}^{N}[P_{n}(x)\left(\pm \left(\frac{(N-n)\kappa_{1}}{4L(n+1)}\right)({\left(x_{\mathrm{edge}}-x\right)}^{2}-L^{2}\right)\\ & \;-\left(\frac{n\;\kappa_{2}}{n-1}\right)J_{\pm }(y|x,n,N))]+{\bar{k}}_{3}\;\Delta x, \end{array}$$

where the choice of ± depends on whether we are considering the right (+) or left (−) edge of the substrate, resulting in $J_{+}(y|x,n,N)=\int^{x_{\mathrm{edge}}}(y-x)P_{b}(y|x,n)\;dy$ and $J_{-}(y|x,n,N)=\int_{x_{\mathrm{edge}}}(y-x)P_{b}(y|x,n)\;dy$. In this case, the bound leg distributions *P*_*b*_(*y*|*x*, *n*) will be skewed in the direction of the substrate, meaning that the contribution to the effective velocity in equation (2.10) due to unbinding events will always act away from the substrate. By contrast, the binding event contribution to the effective velocity in equation (2.10) always acts towards the substrate. Since the effective velocity due to binding events is expected to dominate over the component resulting from unbinding events (see electronic supplementary material, figures S5 and S6*c*), cargo will exhibit an effective velocity that tries to maximize their overlap with the substrate. As a result, cargo can exhibit a stable fixed point in their motion near the edge of the substrate when ${\bar{k}}_{3}\Delta x\neq 0$. This effect has been observed experimentally for actin filaments that interact with microtubules [5].

### 2.4. Using experimentally derived input parameters

**Figure 5.**
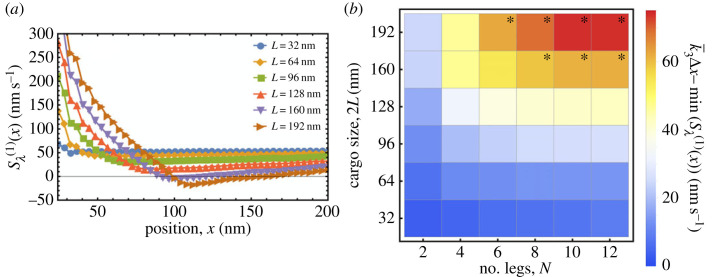
Dynamics of cargo simulated using experimental data. (*a*) The position-dependent effective velocity component $S_{\lambda }^{\left(1\right)}(x)$ exhibited by bound cargo permanently associated with *N* = 10 EBs obtained from stochastic cargo binding simulations (5 × 10^4^ simulated) using input parameters derived from previously published experimental data (see electronic supplementary material, tables S1 and S2). The microtubule edge is set at *x*_edge_ = 0 nm, and stable fixed points are exhibited by 10-legged cargo of sizes 2*L* ≥ 160 nm. Error bars are too small to see. (*b*) A heatmap showing the maximum effective velocity generated by binding or unbinding events in the direction of the growing microtubule end (100 000 simulated for 2 ≤ *N* ≤ 8; 50 000 simulated for *N* = 10; 25 000 simulated for *N* = 12). Cargo can co-move with growing microtubule ends (asterisks) when ${\bar{k}}_{3}\;\Delta x-\min(S_{\lambda }^{\left(1\right)}(x))>57\;\mathrm{nm}\;s^{-1}$, which qualitatively corresponds to the effective velocity generated by binding and unbinding events being able to counter-balance the microtubule growth velocity.

Previous studies have shown that beads coated in EB binding domains can track the growing ends of microtubules in the presence of EBs [5,27], but a model that can describe these dynamics has not yet been developed. In order to test whether the model presented in this work can reproduce the dynamics of multivalent cargo in biologically relevant conditions, we first converted the parameters describing EB–microtubule interactions obtained in previous studies [23,27,36–41] into binding/unbinding rate distributions required as inputs for our stochastic cargo binding simulations (see electronic supplementary material, §7 and table S1). Here, we have subsumed the EB layer into an effective distribution of binding/unbinding rates for the multivalent cargo. Considering EBs explicitly does not fundamentally alter the results. Using these rate profiles, our model predicts that stable fixed points will be exhibited in the motion of (*N* ≥ 6)-legged cargo of sufficient size ($S_{\lambda }^{\left(1\right)}(x^{*})=0$), corresponding to them being able to track growing microtubule ends (figure 5). The component of each cargo’s effective velocity $S_{\lambda }^{\left(1\right)}(x)$ due to binding and unbinding events increases monotonically as a function of *N* until it plateaus, as predicted analytically in §2.3, and increases approximately quadratically as a function of *L* for comparatively small cargo, as predicted by equation (2.9) (see electronic supplementary material, figure S8).

### 2.5. Extending the cargo binding model to two dimensions

The cargo binding model defined in equations (2.1)–(2.5) can be extended to two dimensions to describe more complex cargo–substrate interaction networks. The PDF *P*(**x**, *t*) describing the probability of finding cargo at position **x** = (*x*, *y*) at time *t* can be derived using the same method as in the one-dimensional case (see electronic supplementary material, §8), and the resulting Fokker–Planck equation reads

2.11 $$\begin{array}{rl} \frac{\partial P(x,t)}{\partial t} & =\nabla \cdot \left[\frac{1}{2}\left(\begin{array}{c} S_{\lambda }^{\left(2,x\right)}(x)\;\partial_{x}+S_{\lambda }^{\left(1,xy\right)}(x)\;\partial_{y}\\ S_{\lambda }^{\left(2,y\right)}(x)\;\partial_{y}+S_{\lambda }^{\left(1,xy\right)}(x)\;\partial_{x} \end{array}\right)P(x,t)-v_{\mathrm{eff}}(x)P(x,t)\right]\\ & \;+k_{\mathrm{on}}(x)-k_{\mathrm{off}}(x) \end{array}$$

and

2.12 $$v_{\mathrm{eff}}(x)=\left(\begin{array}{c} S_{\lambda }^{\left(1,x\right)}(x)-\frac{1}{2}(\partial_{x}S_{\lambda }^{\left(2,x\right)}(x)+\partial_{y}S_{\lambda }^{\left(1,xy\right)}(x))\\ S_{\lambda }^{\left(1,y\right)}(x)-\frac{1}{2}(\partial_{x}S_{\lambda }^{\left(1,xy\right)}(x)+\partial_{y}S_{\lambda }^{\left(2,y\right)}(x)) \end{array}\right).$$

Here $S_{\lambda }^{\left(i,\cdot \right)}(x)$ and $\lambda_{m}^{\left(i,\cdot \right)}(x,n)$ are defined in the same way as their one-dimensional counterparts but with spatial integrals running over two dimensions, and $S_{\lambda }^{\left(i,xy\right)}(x)$ and $\lambda_{m}^{\left(i,xy\right)}(x,n)$ are functions of the two-dimensional cross moment $\int dx\int dy({\left[\Delta_{m}^{2}(x-x_{a}(t))(y-y_{a}(t))\right]}^{i}k_{m}(x|x_{a}(t),n))$.

**Figure 6.**
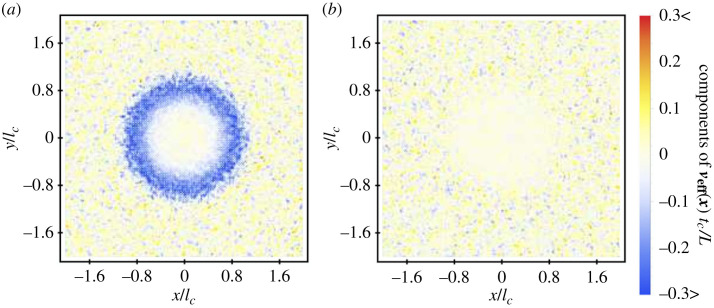
Radial dynamics of cargo in two dimensions. Position-dependent components of the effective velocity in the (*a*) radial direction and (*b*) tangential direction, derived by substituting the results of two-dimensional simulations (2.5 × 10^5^ simulated) into equation (2.12) and applying a change in coordinate systems (colour bar applies to both plots). The components of the effective velocity in Cartesian coordinates are shown in electronic supplementary material, figure S11. Data were averaged over each local point and its four nearest neighbours before changing coordinate systems. The effective velocity results in cargo being ‘attracted’ towards the central region of increased cargo–substrate binding rate defined in equation (2.13).

In a two-dimensional system, cargo legs exhibit the baseline interaction rates *κ*_*m*_(**x**), which must be corrected using a two-dimensional shape factor *S*(**x**|**x**_*a*_(*t*), *n*) and bound leg distribution *P*_*b*_(**x**|**x**_*a*_(*t*), *n*). Once again, the simple shape factor *S*_*H*_(**x**|**x**_*a*_(*t*)) can be introduced that limits the possible binding positions of cargo legs to within the range ∣∣**x** − **x**_*a*_∣∣ ≤ *L*. Then, working through the same procedure followed for the one-dimensional case, we arrive at a complete set of equations for the two-dimensional model (see electronic supplementary material, §8). These equations show that two-dimensional cargo will move diffusively for position-independent binding/unbinding rates (see electronic supplementary material, figure S12*a*), while for spatially dependent rates they will exhibit an effective velocity towards regions of increasing binding rate and decreasing unbinding rate, as in the one-dimensional case. This can be seen, for example, in figure 6, which shows the effective velocity of cargo on a two-dimensional substrate with ${\bar{k}}_{3}=0$, a uniform unbinding rate *κ*_2_, and a non-uniform binding rate defined by

2.13 $$\kappa_{1}(x)=\kappa_{2}\left[\left(\frac{4}{1+\exp({\left(x\cdot x/4\right)}^{3})}\right)+1\right],$$

which is the two-dimensional equivalent of equation (2.8). Similarly to what was observed in the one-dimensional case (figure 3*a*), we see that this binding rate distribution results in an effective velocity for bound cargo that acts purely towards the central region of increased binding rate.

### 2.6. Summary of key points

**Figure 7.**
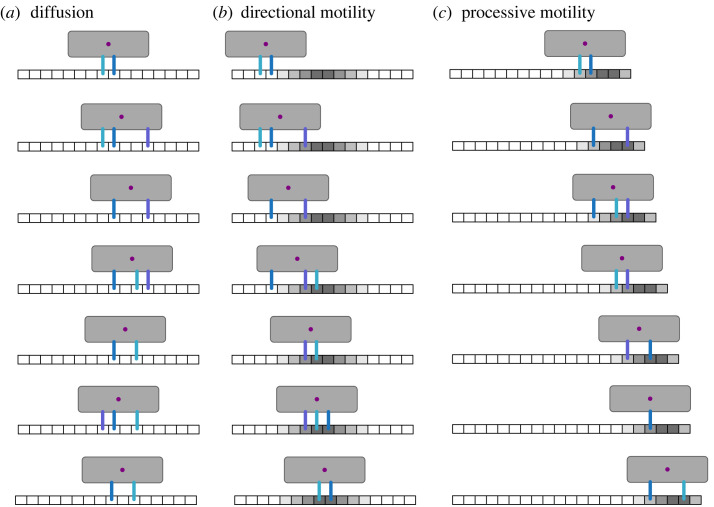
Different types of cargo motion. Schematics showing the three types of motion that the multivalent cargo can display. Increasing grey levels in the substrate sites indicate increasing affinity, i.e. increasing binding and/or decreasing unbinding rate for legs. (*a*) Diffusive motion for substrates with uniform binding and unbinding rates, (*b*) directed motility (on average) towards regions of higher affinity, and (*c*) processive motion of the cargo following the growing substrate edge through preferential unbinding of legs from low-affinity regions and higher probability of rebinding of legs to high-affinity regions on the substrate. Note that (*c*) assumes that the growing edge has a higher affinity than the rest of the substrate.

The model introduced here aims to provide a minimal conceptual framework to understand the noisy dynamics of a cargo interacting with a substrate through the binding and unbinding of its multiple interaction sites, here referred to as legs. We sought an approach that could connect these individual interaction events to the collective motion of the entire cargo. Cargo–substrate interactions begin with the binding of a single leg onto the substrate and end when the last remaining leg unbinds (figure 1). During this time, the position of the cargo is dictated by the average position of its bound legs (figure 1). For homogeneous substrates, this simple dynamics gives rise to an effective diffusion of the cargo with distinct short- and long-timescale diffusivities (figures 2*b* and 7*a*). The difference results from the time required for a cargo to equilibrate the initial distribution of its bound legs to the long-timescale one (see §2.2). We propose that this could explain previous reports of decaying cargo diffusivity at long timescales [5].

Substrates with spatially varying binding/unbinding rates, instead, will give rise to an effective diffusivity *D*_eff_(*x*) and an effective velocity *v*_eff_(*x*) dependent on position. These can be connected to the binding/unbinding rate distributions through the analytical expressions in equations (2.4) and (2.5) (figure 7*b*). The drift is caused by differences in the binding/unbinding rates experienced by different parts of a cargo, leading it to accumulate towards regions of higher binding rates and/or lower unbinding rates (figure 4*a*). Differences in binding and unbinding rates can alternatively be understood as differences in a putative free energy landscape, such that higher binding rates correspond to lower free energies and a stronger ‘attraction’. For a fixed substrate, the system then evolves towards a final steady-state distribution akin to that of a free energy minimum (see for example figure 4*a*), in a way that is conceptually similar to a Brownian particle drifting down a potential well. However, for substrates that grow continuously such as a polymerizing microtubule, the system is kept far from equilibrium by the constant addition of new binding sites and the maturation of old ones (see electronic supplementary material, §7). In this case, if the spatial gradient of binding/unbinding rates is sufficient given the size of the cargo and the number of its legs (see §2.3), the cargo will be able to ‘surf’ the wave of newly added binding sites and follow the growth of the substrate (see figure 7*c* and §2.4). In the Brownian particle analogy, this is equivalent to the potential well exhibiting a constant drift, which causes the equilibrium position of the Brownian particle to be displaced from the centre of the well.

## 3. Discussion

Several experimental systems of different nature can all be conceptualized as a multivalent cargo moving on a substrate. Here, we introduce a unifying mathematical model that is able to capture the diverse range of phenomena observed experimentally, and which leads to an intuitive analytical formulation of the observed dynamics. The model we propose describes the diffusive motion of cargoes with position-independent cargo–substrate interaction rates [1], the processive motion of cargoes that either interact with moving substrates and/or exhibit position-dependent cargo–substrate interaction rates [5,7,27,28,31], and the relatively rapid motion of cargoes away from the edges of substrates to which they are bound [5,7]. The model is easily extendable to higher valency cargo and to systems with more complex interaction networks. In this work, equations that describe cargo motion have been derived explicitly from basic principles involving binding and unbinding events, and it has been shown that gradients in cargo–substrate interaction rates govern the direction and magnitude of the effective velocity exhibited by cargo.

In the case of microtubule tip-tracking, using as inputs only the binding and unbinding rate distributions of EBs derived from previous experiments, the model presented here was able to qualitatively reproduce experimentally observed phenomena without any further parameter fitting. Previous studies have estimated the effective forces acting on cargo that are required to generate an effective velocity and diffusivity using the formula *F*(*x*) ≃ (*k*_*B*_ *T* *v*_eff_(*x*))/*D*_eff_(*x*) [5]. At the stable fixed points of cargo motion in figure 5*a*, this method predicts forces of approximately 0.1 pN (assuming a temperature of 37°C), which is of the same order of magnitude as those obtained from optical tweezer experiments [5,27], and approximately an order of magnitude smaller than those generated by individual motor proteins [42]. Assuming that motor proteins travel at velocities roughly an order of magnitude greater than the average microtubule growth velocity (the velocity exhibited by cargo while co-moving with the growing microtubule end) [40,43–45], and that the effective drag coefficients of cargo scale ∝*r*_cargo_ (in accordance with Stokes’ drag force), it can be predicted that the maximum size of cargo that can be transported by EB-mediated mechanisms is $r_{\mathrm{tip}}^{\max}=(F_{\mathrm{tip}}^{\max}/F_{\mathrm{motor}}^{\max})(v_{\mathrm{motor}}^{\max}/v_{\mathrm{tip}}^{\max})r_{\mathrm{motor}}^{\max}∼r_{\mathrm{motor}}^{\max}$. This simple estimate suggests that, although EB-mediated cargo transport is slower than transport using motor proteins, the maximum size of cargo that can be transported by both mechanisms is approximately the same. The sizes of cargo shown to track growing microtubule ends in figure 5 are similar to the size of beads previously shown to exhibit these dynamics in experiments [5,27]. The latter can have effective diameters of up to approximately 60−70 nm after considering the sizes of EB–cargo linkers (approx. 10 nm) and EBs (approx. 13.6 nm maximum diameter; see electronic supplementary material, table S1 [37]). Unlike for previously published experimental data, where it was not possible to predict the number of cargo-associated EBs that could interact with microtubules, figure 5 suggests that a surprisingly small number of permanently associated EBs (approx. 8) is required to sustain cargo transport. The steep velocity profile observed in figure 5*a* at positions close to the edge of the microtubule (*x* = 0 nm) is generated by the same mechanism as the effective velocity in equation (2.10).

It is possible that steric effects arising from the tethering of EBs to cargo and cargo to microtubules could alter the binding and unbinding rates defined in electronic supplementary material, tables S1 and S2. This model allows these effects to be studied mathematically, either by introducing cooperativity into the binding dynamics of cargo legs, or by increasing the complexity of the shape factor used in continuum-level calculations. It is also possible that larger cargoes will break the assumption that the timescale associated with the diffusion of unbound legs on the cargo surface is much smaller than the legs’ typical binding timescale with the substrate (*t*_*d*_ ≪ *t*_*b*_). This would have the effect of introducing an explicit time dependence in the shape factor for each leg, which may inhibit the diffusive and processive motion of the whole cargo. For EB-mediated cargo dynamics, the extension of the model to two dimensions allows for the implementation of a two-dimensional shape factor that takes into account the curvature of the cylindrical microtubule substrate. The two-dimensional model could also provide a novel route for studying cell locomotion, where binding and unbinding events will correspond to tethering and untethering events between a cell and a substrate (respectively). In this case, this work could provide an intuitive model for studying the dynamics of cells exhibiting chemotaxis or haptotaxis.

This work builds upon the results of previously published models for multivalent cargo dynamics by bridging between the discrete binding/unbinding kinetics at individual interaction sites and the resulting motion of a generic multivalent cargo. By introducing additional types of interactions to the three defined in §2.1 (binding/unbinding to/from the substrate and the effects of deterministic cargo/substrate velocity), the model described in this work can also be used to probe more complex cargo dynamics. This could include the effects of competition for binding sites between cargoes, or the effects of introducing additional molecules that can bind to the cargo or its legs and inhibit their ability to bind to the substrate [33]. We hope that our results will inspire new applications within the many areas of biophysics research where protein or cell motion is the result of complex networks of binding dynamics.

## 4. Material and methods

### 4.1. Simulation methods

Stochastic cargo binding simulations were implemented in Matlab using the Gillespie algorithm [46,47] to probe the system state in continuous time (see electronic supplementary material, §9). Molecular dynamics simulations were also implemented in Matlab, but instead updated cargo positions according to the Langevin equation defined in equation (2.6) using a forwards Euler scheme. Wiener process displacements were calculated using inverse transform sampling and binding dynamics were introduced by randomizing the position of the cargo within the periodic domain with rate *k*_ran_(*x*) = 1/*t*_dwell_(*x*) (calculated by substituting the local binding and unbinding rates into a previously published average dwell time formula [29,30]).

### 4.2. Numerical methods

Numerical solutions to the Fokker–Planck equation defined in equation (2.3) were obtained using the built-in Matlab function pdepe().

## Data Availability

All of the data are derived from codes available from the Zenodo repository: https://www.doi.org/10.5281/zenodo.8289675 [48]. Supplementary material is available online [49].
